# Enhanced Activation of Motor Execution Networks Using Action Observation Combined with Imagination of Lower Limb Movements

**DOI:** 10.1371/journal.pone.0072403

**Published:** 2013-08-28

**Authors:** Michael Villiger, Natalia Estévez, Marie-Claude Hepp-Reymond, Daniel Kiper, Spyros S. Kollias, Kynan Eng, Sabina Hotz-Boendermaker

**Affiliations:** 1 Spinal Cord Injury Center, University Hospital Balgrist, Zurich, Switzerland; 2 Institute of Neuroradiology, University Hospital Zurich, Zurich, Switzerland; 3 Institute of Neuroinformatics, University of Zurich and ETH Zurich, Zurich, Switzerland; University of Bologna, Italy

## Abstract

The combination of first-person observation and motor imagery, i.e. first-person observation of limbs with online motor imagination, is commonly used in interactive 3D computer gaming and in some movie scenes. These scenarios are designed to induce a cognitive process in which a subject imagines himself/herself acting as the agent in the displayed movement situation. Despite the ubiquity of this type of interaction and its therapeutic potential, its relationship to passive observation and imitation during observation has not been directly studied using an interactive paradigm. In the present study we show activation resulting from observation, coupled with online imagination and with online imitation of a goal-directed lower limb movement using functional MRI (fMRI) in a mixed block/event-related design. Healthy volunteers viewed a video (first-person perspective) of a foot kicking a ball. They were instructed to observe-only the action (O), observe and simultaneously imagine performing the action (O-MI), or imitate the action (O-IMIT). We found that when O-MI was compared to O, activation was enhanced in the ventralpremotor cortex bilaterally, left inferior parietal lobule and left insula. The O-MI and O-IMIT conditions shared many activation foci in motor relevant areas as confirmed by conjunction analysis. These results show that (i) combining observation with motor imagery (O-MI) enhances activation compared to observation-only (O) in the relevant foot motor network and in regions responsible for attention, for control of goal-directed movements and for the awareness of causing an action, and (ii) it is possible to extensively activate the motor execution network using O-MI, even in the absence of overt movement. Our results may have implications for the development of novel virtual reality interactions for neurorehabilitation interventions and other applications involving training of motor tasks.

## Introduction

Over the last two decades several research groups have published data lending support to the “simulation or resonance theory of action” hypothesis formulated by Jeannerod [1]. According to this theory, observing, imagining, and even understanding motor actions activate the neural network involved in motor execution. Although these states differ from one another, there is a partial overlap between covert and overt actions. Mental practice is an accepted training method to improve performance in sports and rehabilitation [2]. Most experiments to date have focused on the upper limbs and investigated either observation or motor imagery, but not the simultaneous combination of both [3].

Recently, there has been a larger increase in the number of situations where people can engage in combinations of observation and motor imagery, i.e. a cognitive process in which a subject imagines himself/herself in the displayed movement situation. People do this mainly while playing “first-person shooter” computer games, while watching point-of-view (POV) scenes in some movies and while undergoing neurorehabilitation (for a review see [4], [5]). With respect to neurorehabilitation, the presentation of limbs aim to (re)activate brain functions that have been abolished due to cortical or subcortical injury (e.g. [6], [7]). The visual stimuli incorporated in the environment guide the motor simulation [8] and might support people who are not able to rehearse motor tasks extended periods of time [9].

To our knowledge, only four studies investigated such combinations of observation and motor imagery with functional MRI (fMRI). In the study by Cross et al. [8] dancers observed and mentally simulated another dancer’s movements; the experimenters found enhanced activation in brain regions classically associated with both action simulation and action observation. Macuga and Frey [10] showed that the observation of intransitive thumb-finger movements increased activation in a subset of the brain areas engaged during observation combined with imagination. In the recent study by Nedelko et al. [2] brain activation of healthy subjects was investigated during action observation alone and during action observation with additional action imagery of video clips showing simple, object-related hand actions. They concluded that both conditions produced similar activation patterns with more activation with action imagery. With respect to lower limb movements, investigations on observation or motor imagery are quite sparse. Brain activity elicited by observation with simultaneous, i.e. “online”, motor imagery of foot movements have been reported previously for gait imagination during observation using different external cues [11]. Table 1 summarizes the results for toe/foot movement experiments that focused on at least one of the above-mentioned activities [12], [13], [14], [15], [16], [17], [18], [19], [20], [21], [22], [23], [24], [25], [26], [27], [28], [29], [30], [31], [32], [33], [34], [35], [36]. According to Jackson et al. [24] the first-person (kinesthetic) perspective recruited the motor execution network more extensively than the third-person (visual) view while watching video clips of hand or foot movements. Regarding motor imagery, the subject is a performer (internal imagery), whereas in the third-person view the subject is a spectator (external imagery) [37]. Most investigations on motor imagery of foot movements have supported this concept [12], [19], [20], [22], [23], [27], [31], [34]. Thus, in the current study, a first-person perspective was chosen.

**Table 1:** fMRI/PET papers with (not) goal-directed toe/foot movements during observation, imagination and imitation.

Paper	Action	Observation	Imagination	Imitation/Execution	Stimulus	Mode	Subjects
Alkadhi et al., 2005	not goal-directed	no	yes (1st)	no/yes	Foot movements	fMRI	8 SCIs/8 Controls
Aziz-Zadeh et al., 2006	goal-directed	yes (3rd)	no	no/no	Foot pressing on objects	fMRI	12
Buccino et al., 2001	goal-directed	yes (3rd)	no	no/no	Foot pressing onobjects/kicking a ball	fMRI	12
	not goal-directed	yes (3rd)	no	no/no	Foot mimicking object actions	fMRI	12
Catmur et al., 2008	not goal-directed	yes (3rd-sagittal)	no	no/yes	Foot movements	fMRI	20
Chaminade et al., 2005	not goal-directed	no	no	yes, online (1st)/no	Foot movements	fMRI	12
Cramer et al., 2005	goal-directed	no	yes (3rd-sagittal)	no/no	Foot pressing on objects	fMRI	11 SCIs/12 Controls
Cramer et al., 2007	goal-directed	no	yes (3rd-sagittal)	no/no	Foot pressing on objects	fMRI	10 SCIs/10 Controls
Ehrsson et al., 2003	not goal-directed	no	yes (1st)	no/yes	Toe movements	fMRI	7
Enzinger et al., 2008	not goal-directed	no	yes (1st)	no/yes (also passive)	Foot movements	fMRI	1 SCI/5 Controls
Gustin et al., 2010	goal-directed	no	yes (1st)	no/no	Foot pressing on car accelerator	fMRI	11 SCIs/19 Controls
Hotz-Boendermakeret al., 2008	not goal-directed	no	yes (1st)	no/yes	Foot movements	fMRI	9 SCIs/12 Controls
Hotz-Boendermakeret al., 2011	not goal-directed	yes (3rd-sagittal)	yes (1st)	no/yes	Foot movements	fMRI	9 SCIs/12 Controls
Jackson et al., 2006	not goal-directed	yes (1st/3rd)	no	yes, online (1st/3rd)/no	Foot movements	fMRI	16
Jastorff et al., 2010	goal-directed	yes (3rd)	no	no/no	Foot dragging, dropping, grasping, pushing obj.	fMRI	18
Jastorff et al., 2012	goal-directed	yes (3rd)	no	no/yes	Foot dragging, dropping, grasping, pushing obj.	fMRI	15
Lafleur et al., 2002	not goal-directed	no	yes (1st)	no/yes	Foot movements	PET	9
Lee et al., 2009	not goal-directed	no	yes (1st)	no/no	Toe movements	fMRI	5
Orr et al., 2008	goal-directed	yes (3rd-sagittal)	yes (3rd-sagittal)	no/yes	Foot pressing on objects	fMRI	10
Rocca and Filippi, 2010	not goal-directed	yes (3rd)	no	no/yes	Foot movements	fMRI	21
Roux et al., 2003	not goal-directed	no	yes (1st)	no/yes	Toe movements	fMRI/PET	10 Amputees/10 Controls
Sahyoun et al., 2004	not goal-directed	no	yes, preparation (1st)	no/yes (also passive)	Foot movements	fMRI	12
Sakreida et al., 2005	not goal-directed	yes (3rd-sagittal)	no	no/no	Foot movements	fMRI	19
Stippich et al., 2002	not goal-directed	no	yes (1st)	no/yes	Toe movements	fMRI	14
Wang et al., 2008	not goal-directed	no	yes, online (1st)	no/yes	Foot/leg movements	fMRI	14
Wheaton et al., 2007	not goal-directed	yes (3rd)	no	no/no	Foot/leg movements	fMRI	12
Yuan et al., 2010	not goal-directed	no	yes (1st)	no/yes	Foot movements	fMRI	13
**Villiger et al.**	goal-directed	yes (1st)	yes, online (1st)	yes, online (1st)/no	Foot kicking a ball	fMRI	12

Papers only with pure execution are not listed and the viewpoint (1st- or 3rd-person) is labeled in brackets.

In our own previous studies, we showed that during motor imagery alone, brain areas in the neural motor network involving pre- and supplementary motor areas, PM cortex, the parietal cortical lobules and prefrontal areas were engaged [12], [22]. In addition, other studies reported M1/S1 activation [19], [29].

In the present investigation, we apply the combination of observation and motor imagery, which we call “observation with online motor imagination”, of a simple transitive foot movement. To identify and control the neural activation specific to online motor imagination, an online imitation condition, as in Jackson et al. [24], was used in this study. We thus investigated brain activity during goal-directed lower limb movements presented from the first-person perspective, during observation-only (O), online motor imagination (O-MI) and online imitation (O-IMIT). We predict that observation combined with imagination of the displayed lower limb movements (O-MI) would potentiate the activation of areas responsive to motor observation and thus induce broader and greater activation than observation-only (O). In addition, we predict that O-MI would activate the motor execution network.

## Materials and Methods

### Participants

Informed written consent was obtained from all subjects and the experimental protocol was in accordance with the Declaration of Helsinki and performed with the approval of the Cantonal Ethics Committee at University Hospital Zurich (EK-24/2009). Fourteen healthy volunteers participated (mean age 25 years, range 18–29 years, 6 females). They had normal or corrected-to-normal visual acuity, no history of psychiatric or neurological disorder and were right-footed (preferred kicking foot).

### Stimuli and Task

**Figure 1:**
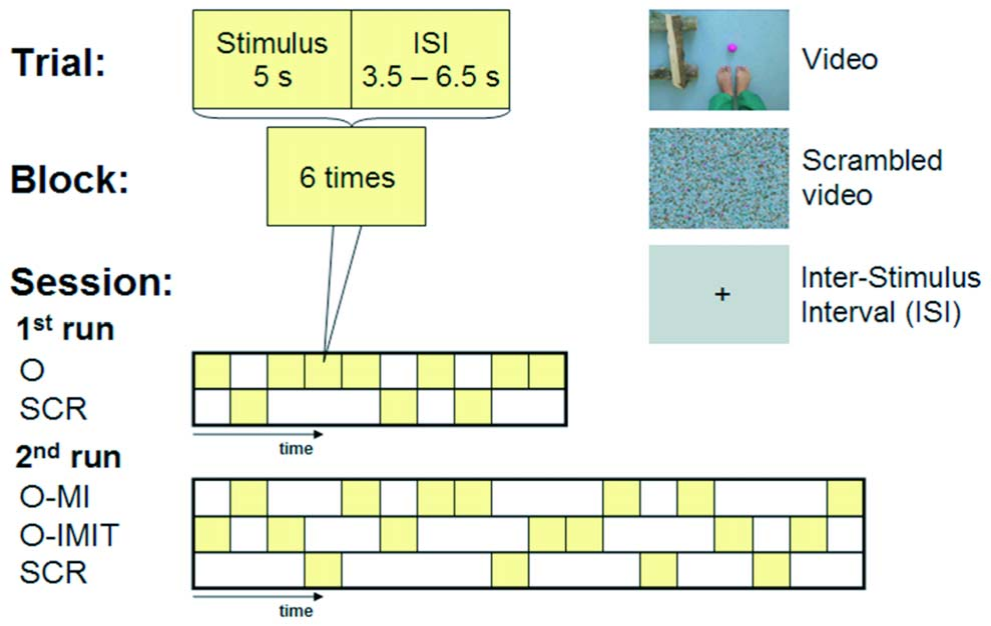
fMRI design. The session consisted of 2 runs containing a total of 7 blocks of each of the 4 conditions. Each block contained 6 trials of the same condition. Each block was preceded by the presentation (1.5 sec) of an instruction. Within a run the blocks were presented in pseudo-random order. Each trial consisted of a 5 s video clip followed by an inter-stimulus interval (ISI) with a duration jittered between 3.5 and 6.5 s. In the first run only the O and SCR conditions were included. The second run included blocks of O-MI, O-IMIT and SCR. The protocol was conceived to yield the same number of trials in each condition (42).

The stimulus was a 5 s video clip showing a first-person perspective view, i.e. looking down on the feet, of a right foot kicking a ball towards a wooden goal (Figure 1). At the start of the video clip both feet were together on the ground. After 0.75 s the right foot lifted and moved forwards towards the ball, kicked it sideways into the goal, and returned to the starting standing position. The movement phase lasted about 3.5 s. A scrambled version of the video clip (Figure 1) was used as a control (baseline) for low-level visual perception [38]. The following four conditions were investigated:

1. Observation-only (O): the subjects had to carefully observe the video clip showing the goal-directed foot movement. The instruction used was: *‘Please look carefully at the video’.*
2. Online motor imagination (O-MI): the subjects observed the video displaying the goal-directed foot movement and had to imagine themselves performing the movement at the same time, i.e. online. The instruction was: ‘*When you see the foot moving in the video, start immediately to imagine that the presented moving foot is yours and try to control the movement in your mind by continuously watching the video’.*
3. Online imitation (O-IMIT): the subjects executed a right-foot dorsiflexion, followed by a movement like a ‘windshield wiper’ going from the right to the left and back to the starting position. The instruction was: ‘*When you see the foot moving on the video, start immediately to perform the presented movement with your own foot and keep watching the video’.*
4. Scrambled video clip as baseline (SCR): the subjects had to carefully observe the scrambled video clip after receiving the same instruction as in the O condition.

The subjects’ behavior was monitored and controlled for immobility with a video camera in the O, O-MI and SCR conditions and for correct performance during O-IMIT.

### Neuroimaging and Behavior

Before the scanning session, subjects received verbal and written information about the experiment and practiced the tasks. Both the 5 s video clip and its scrambled version were presented outside the scanner. Without mentioning the O-MI task, the O and SCR conditions were presented. The instruction was given for the O-IMIT task and the required movement was practiced until correctly performed.

The fMRI session consisted of 2 runs, each containing 7 blocks of 6 trials of the same condition. Each block was preceded by 1.5 s written instruction (‘observe’, ‘observe and imagine’, ‘observe and imitate’). The blocks were presented within the run in pseudo-random order (Figure 1). One trial consisted of a 5 s video clip followed by an inter-stimulus interval (ISI) lasting between 3.5 and 6.5 s. The ISI was a grey screen with a fixation cross.

In the first run only O and SCR were included, to avoid that the two active conditions (O-MI and O-IMIT) interfere with O. This run lasted 10 min 15 s and was followed by a rest period in which subjects received verbal information about the next tasks. This information was as follows: ‘*While the video clip is presented, there are two new tasks, one is to imitate the movement online (‘observe and imitate’) and the other to imagine the movement online (‘observe and imagine’). Before the task, you will get a written instruction on the task you should perform. After the instruction ‘observe and imagine’ you imagine that the presented foot is yours and you perform the movement in your mind, while watching the video. During the task ‘observe and imitate’ you perform the foot movement you exercised before’.* This second run included pseudo-randomly interleaved blocks of O-MI, O-IMIT and SCR and lasted 18 min 27 s (Figure 1).

This protocol was designed to yield the same number of trials (42) in each condition. For the SCR condition, this number was achieved by cumulating the blocks of the first and second runs. The video was presented on a rear-projected screen located inside the scanner room, approximately at the level of subject’s feet. The participants could see the screen via a mirror attached to the head coil, and the legs and head were stabilized to minimize movement artifacts. Subjects performed the tasks with their shoes off and their legs slightly raised and supported by pillows. Sandbags were placed on both legs in order to limit leg movements. In order to reduce head motion artifacts during the data acquisition, we used a custom-made head support which covered the superior and partially the lateral parts of the subjects head. Furthermore, foam pillows were used to additionally restrict the motion in the left-right direction [39].

After the scanning session, participants completed the kinesthetic part of the Vividness of Motor Imagery Questionnaire (VMIQ) [40]. In addition, they rated their subjective ability to mentally perform the foot movement using a 5-point rating scale (from 1 =  best to 5 =  worst).

To check for muscle inactivity during observed and imagined movements a mock-up of the scanner was used. EMG was recorded in four randomly chosen subjects after the scanning using dual surface electrodes (Noraxon, Cologne, Germany) placed on the right anterior tibialis muscle. The subjects were lying supine as they had been in the scanner. The EMG signals during the movement phase in the video (3.5 s) were amplified, band-pass filtered (10–500 Hz) and rectified. All signals were sampled at 1500 Hz and the muscle activity during the four conditions was analyzed by calculating the root mean square (RMS). The RMS values of the EMG responses of the conditions were compared to each other using an ANOVA with post-hoc Bonferroni correction.

### Neuroimaging Data Acquisition

The functional images were measured with T2*-weighted echo-planar images (EPIs) using blood-oxygenation-level-dependent (BOLD) contrast on a 3-T, whole-body, MRI scanner (Philips Medical Systems, Eindhoven, The Netherlands), equipped with an 8 channel SENSE™ head coil. The stimulus presentation was controlled and synchronized with the fMRI scanning using *Presentation* (Neurobehavioral Systems Inc., Albany, CA, USA),

The image acquisition parameters were as follows: repetition time (TR) = 3 s, echo time (TE) = 35 ms, flip angle (FA) = 82°, field of view (FOV) = 220 mm, matrix size = 80×80, 45 slices with 3 mm thickness without gap, voxel size = 2.75×2.75×3 mm. Additionally, high-resolution whole brain images were acquired from each participant using the 3D T1W TFE scan: TR = 20 ms, TE = 4.6 ms, FA = 20°, FOV = 220 mm, 210 slices with 0.75 thickness, voxel size = 0.98×0.98×0.75. For the functional run, the first five images were always discarded to allow for signal stabilization. For the 1^st^ run 205 volumes were collected and stored and 369 volumes were stored for the 2^nd^ run.

### Neuroimaging Preprocessing and Data Analysis

All fMRI analyses were performed using SPM5 (Welcome Department of Imaging Neuroscience, London, UK; http://www.fil.ion.ucl.ac.uk/spm/). Functional images from each subject were realigned, spatially normalized into the Montreal Neurological Institute (MNI) space with a resolution of 2×2×2 mm and then smoothed with a 6 mm full-width at half-maximum (FWHM) Gaussian kernel. For removing the low frequency noise, a high-pass filter with a cut-off of 128 s was used. Data were analyzed using a random-effect model to allow for population inferences [41]. The general linear model (GLM) was fitted for each subject by a design matrix comprising the onsets and durations of each condition (video) and convolved with the standard canonical hemodynamic response function. The four conditions described previously were included in the model. Six regressors (of no interest) were incorporated to account for rigid-body movement effects. The data used originate from the realign job with SPM (time series of translations in the x, y and z direction and rotations about the x, y and z axes) to discount movement effects when looking for brain activations [42]. Respective parameter estimates (beta) and contrast images (cons) were computed by voxelwise comparisons.

To determine the group activation in the three experimental conditions (O, O-MI and O-IMIT) and for the baseline (SCR), the single-subjects contrasts were entered into a second-level analysis for each of the contrasts. All neuroimaging analyses were evaluated in whole-brain analyses at a voxel-wise threshold of p<0.001 uncorrected. Because clusters of systematically increasing size are less probable, a spatial extent threshold can be determined where clusters of a greater size occur less frequently. After running 10000 iterations with a Monte Carlo simulation (http://afni.nimh.nih.gov/pub/dist/doc/program_help/AlphaSim.html), a cluster-extent threshold of 31 contiguous voxels was necessary to correct for multiple comparisons and achieve a significance level of p<0.05 for a voxel threshold of p<0.001 [43], [44]. Thus, only clusters of activation meeting or exceeding that size were listed in the tables of the present study [45]. Furthermore, corrections for multiple comparisons were additionally performed with a FWE cluster-corrected level of p<0.05. In the Discussion section, we focus on brain regions that reached FWE cluster-corrected significance.

The designed neuroimaging analyses were used to achieve four objectives:

First, contrasts were used to test for differences between each of the experimental conditions (O, O-MI and O-IMIT) and the baseline (SCR) using one-sample t-tests.

Second, contrasts were also used to test for differences between conditions (O vs O-MI and vice-versa, O vs O-IMIT and vice-versa, and O-MI vs O-IMIT and vice-versa) using paired t-tests.

Third, a flexible full factorial ANOVA was conducted to determine areas of overlapping brain regions (conjunction null method) [46].

Fourth, ANOVAs were conducted to test for mean percent signal change differences of the BOLD responses (± standard error of the mean [SEM] across subjects) between conditions (O, O-MI and O-IMIT) within selected conjunction activated regions. For post-hoc pairwise comparisons, Bonferroni adjusted p-values were used.

All imaging results were displayed on either rendered cortical surface or on slices of a high-resolution structural MRI scan of a standard brain from the MNI. Anatomical identification was performed with the WFU PickAtlas (Wake Forest University, Winston-Salem, NC, v2.4) and the included Anatomic Automatic Labeling (AAL) atlas [47], [48]. The cortical identified regions included the paracentral lobule (M1/S1), the pre- and supplementary motor area (preSMA, SMA), cingulate gyrus (CG), precentral gyrus and frontal operculum (PMd and PMv), superior parietal lobule (SPL), inferior parietal lobule (IPL) and precuneus (PCu), prefrontal cortex (PFC), insula (INS), hippocampus (HC), occipitotemporal cortex (OTC), and subcortically, the thalamus (THAL), putamen (PUT), caudate nucleus (CN), and cerebellum (CB–peak location based on Schmahmann et al. [49]).

## Results

### Imaging Results

#### 1. Comparison between experimental conditions and baseline - effects of condition

The group results of the O, O-MI and O-IMIT conditions contrasted with the SCR condition (baseline) are summarized in Table 2. Overall, from O to O-MI to O-IMIT more brain regions were activated and the cluster size increased. More detailed comparisons are described in the following sections.

**Table 2:** MNI coordinates for group activations for observation-only (O), online motor imagination (O-MI) and online imitation (O-IMIT) versus the baseline (SCR) condition.

Region	Left/Right	O>SCR			O-MI>SCR			O-IMIT>SCR		
		x y z	t- value	Vol.	x y z	t -value	Vol.	x y z	t -value	Vol.
Paracentral lobule (M1/S1)	L							**−2 −26 66**	***5.21***	**78**
Supplementary motor area (SMA)	L/R							**−6 −12 6**	***8.36***	**348**
Presupplementary motorarea (preSMA)	L/R				−6 14 44	*6.81*	31	**6 4 46**	***11.35***	**1521**
Ventral premotor cortex (PMv)	L				**−52 6 2**	***6.47***	**125**	**−50 6 2**	***7.68***	**423**
	R				**58 8 4**	***6.02***	**64**	**60 8 2**	***6.53***	**60**
Precuneus (PCu)	L/R	**6 −66 32**	***7.97***	**1040**	**12 −62 56**	***7.12***	**103**	**−8 −60 64**	***8.80***	**676**
Inferior parietal lobule (IPL)	L	**−56 −30 24**	***7.03***	**122**	**−48 −36 34**	***6.26***	**287**	**−60 −24 12**	***9.68***	**775**
	R				**62 −32 22**	***5.53***	**116**	**58 −32 30**	***7.35***	**915**
Occipitotemporal cortex (OTC)	L	**−46 −80 6**	***7.70***	**543**	**−54 −68 2**	***8.76***	**553**	**−54 −72 0**	***10.41***	**634**
	R	**42 −74 −2**	***8.13***	**626**	**44 −76 0**	***7.86***	**533**	**46 −76 2**	***12.12***	**959**
Insula (INS)	L				−34 10 8	*5.40*	34			
	R							**40 −12 −6**	***7.58***	**138**
Thalamus (THAL)	L	**−14 −24 14**	***5.51***	**83**				**−20 −24 10**	***14.05***	**1939**
Putamen (PUT)	L				**−22 0 12**	***7.68***	**239**			
Cerebellum (CB) Lobule VI	L				**−8 −70 −10**	***6.56***	**276**	**−18 −74 −22**	***6.86***	**90**
Cerebellum (CB) Crus I	L	**−2 −78 −22**	***6.60***	**54**				**−38 −54 −34**	***7.34***	**97**
	R	**40 −56 −28**	***6.19***	**82**	**38 −60 −28**	***11.08***	**131**	**40 −58 −30**	***10.59***	**378**

Results were calculated at a voxel-threshold of p<0.001 (uncorrected) with a spatial extent of k≥31 voxels. Entries in bold denote activations significant at the FWE cluster-corrected level of p<0.05.

The **O condition** activated a region in the medial wall of the parietal lobe identified as the posterior PCu, a multimodal sensory input integration area. Additional foci were found in the left posterior THAL, IPL and bilateral CB (Crus I). Low-level visual activations were excluded by the scrambled baseline, i.e. no V1 activation, but the observation of the foot movements still activated bilaterally the OTC.

During the **O-MI condition** all cortical areas detected in the O condition, i.e. PCu, IPL and OTC, were also activated. Additional activated areas included the PM cortex, involved in motor imagery. Subcortical foci were also found in the left PUT and right (Crus I) and left (lobule VI) CB.Additionally, activations not surviving FWE cluster-correction were observed in the preSMA and left INS.

As expected, the **O-IMIT condition** activated an extensive motor cortical network including the foot representation in the left M1/S1 cortex and SMA. These regions were not activated, neither in the O nor in the O-MI conditions. In addition, O-IMIT showed enlarged clusters in almost all areas found in the O-MI condition, i.e. preSMA, left PMv, IPL and OTC, PCu. Further foci were found in the right INS, left THAL and bilateral CB.

#### 2. Comparison between conditions O, O-MI and O-IMIT

In the second level analysis, the conditions were contrasted with each other to reveal the activations specific to each condition (Table 3).

**Table 3:** MNI coordinates of contrasts for O-MI>O, O-IMIT>O and O-IMIT>O-MI.

Region	Left/Right	O-MI>O			O-IMIT>O			O-IMIT>O-MI		
		x y z	t value	Vol.	x y z	t value	Vol.	x y z	t value	Vol.
Paracentral lobule (M1/S1)	L				**−4 −38 72**	***6.49***	**91**			
Supplementary motor area (SMA)	L/R				**2 −4 50**	***9.61***	**848**	**4 −6 46**	***12.88***	**1504**
Presupplementary motor area (preSMA)	L/R	4 20 46	*5.33*	35	**6 2 46**	***7.08***	**397**			
Dorsal premotor cortex (PMd)	L	−46 −6 52	*5.55*	39						
Ventral premotor cortex (PMv)	L	**−54 2 2**	***6.70***	**361**	**−56 2 2**	***11.40***	**1864**			
	R	**54 4 0**	***7.33***	**111**	**56 6 2**	***8.50***	**285**			
Precuneus (PCu)	L				**−8 −52 72**	***10.86***	**1056**			
Inferior parietal lobule (IPL)	L	**−42 −56 50**	***6.59***	**49**	**−62 −22 14**	***9.57***	**626**			
	R	60 −22 34	*5.53*	34	**62 −22 36**	***12.09***	**1208**	**52 −32 30**	***6.04***	**211**
Insula (INS)	L	**−32 12 6**	***5.57***	**51**						
Putamen (PUT)	L	−30 −10 6	*6.27*	43	**−24 8 6**	***10.20***	**1392**	**−24 8 2**	***12.28***	**415**
	R							**34 0 4**	***9.25***	**522**
Cerebellum (CB) Lobule VI	L				**−22 −72 − 24**	***7.19***	**265**	**−22 −74 −24**	***8.02***	**434**
	R				**32 −56 −30**	**5.65**	**107**			

Results were calculated at a voxel-threshold of p<0.001 (uncorrected) with a spatial extent of k≥31 voxels. Entries in bold denote activations significant at the FWE cluster-corrected level of p<0.05.

The contrasts between the O condition and the two other conditions (**O>O-MI and O>O-IMIT**) did not reveal any significant differences in activation.

**Figure 2:**
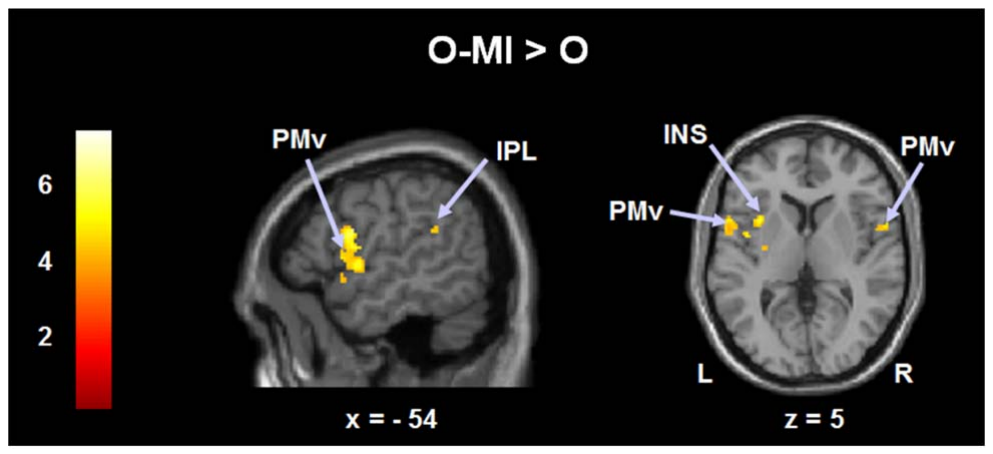
Activation patterns during right foot movements in healthy subjects from the contrast O-MI>O. The results are superimposed on the MNI template and the regions are listed in Table 3. Numbers in the color bar correspond to t-values. Abbreviations: PMv: ventral premotor cortex; IPL: inferior parietal lobe; INS: insula.

The most prominent contrast was between **O-MI and O** which revealed a strong increase in activation in PMv bilaterally, left IPL, and left INS (Figure 2). BOLD signals not surviving FWE cluster-correction were observed in preSMA, left PMd, right IPL and left PUT.

The **O-IMIT>O** contrast revealed regions specific to motor execution, such as M1/S1, preSMA and SMA proper, bilateral PMv and PUT in the left hemisphere, contralateral to the moving right foot. The activation patterns also included foci in the bilateral IPL and in both lobules VI of the CB. Compared to the **O** condition, a more anterior part of the PCu was activated.

The contrast **O-IMIT>O-MI** revealed enhanced activation in the right SMA and IPL, but interestingly not in M1/S1. Additional activations were found in the bilateral PUT and the left CB (lobule VI). The inverse contrast **O-MI>O-IMIT** did not reveal any significant activation changes in cortical and subcortical regions.

#### 3. Conjunction and percent signal change

**Figure 3:**
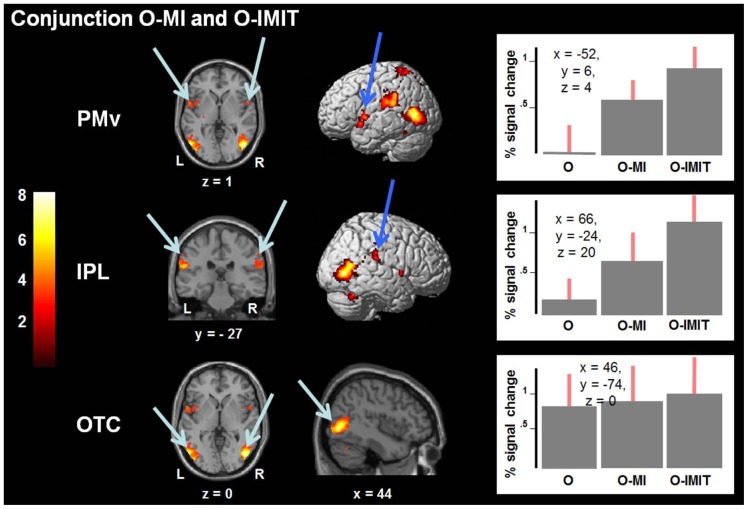
Conjunction (shared activations) and percent signal changes. Conjunction (shared activations) of O-MI and O-IMIT are displayed in the left two columns. The results are superimposed on the MNI template and regions are listed in Table 4. Numbers in the color bar on the left side correspond to t-values. In the right column, percent signal changes of the BOLD responses (± SEM across subjects) for the group local maximum in left PMv, right IPL, and right OTC are shown in each condition (O, O-MI and O-IMIT). Abbreviations: same as Figure 2; OTC: occipitotemporal cortex.

The conjunction of the three experimental conditions taken together (O+O-MI+O-IMIT) revealed strong common bilateral activation in the OTC (left: −54, −72, 0– k = 885; right: 46, −74, 0– k = 913), which was almost equally activated in the three conditions (Figure 3), in SPL (−24, −56, 66– k = 205) and PCu (−6, −60, 52– k = 105) of the left hemiphere.

Pair-wise conjunctions of **O and O-MI,** of **O and O-IMIT** and of **O-MI and O-IMIT** revealed several shared regions of activation. In the conjunctions of **O and O-MI** and of **O and O-IMIT** the same clusters as in the conjunction of all three conditions taken together were activated. In contrast, the conjunction of **O-MI and O-IMIT** revealed shared activation in additional regions, i.e. left PMv, i bilateral IPL and in right CB, with stronger BOLD increase for O-IMIT than O-MI (Figure 3 and Table 4). In addition, activations not surviving FWE cluster-correction were observed in the right PMv, left INS and PUT.

**Table 4:** Conjunction (shared activations) of O-MI and O-IMIT.

Region	Left/Right	Conjunction O-MI and O-IMIT		
		x y z	t-value	Vol.
Ventral premotor cortex (PMv)	L	**−52 6 4**	***5.53***	**313**
	R	56 6 2	*3.80*	40
Precunues (PCu)	L	**−6 −58 52**	***4.46***	**108**
Superior parietal lobule (SPL)	L	**−24 −56 66**	***4.34***	**208**
Inferior parietal lobule (IPL)	L	**−56 −42 32**	***6.17***	**754**
	R	**66 −24 20**	***4.31***	**124**
Occipitotemporal cortex (OTC)	L	**−54 −72 0**	***8.06***	**1004**
	R	**46 −74 0**	***7.66***	**943**
Insula (INS)	L	−40 8 2	*4.03*	45
Putamen (PUT)	L	−22 2 14	*4.26*	51
Cerebellum (CB) - Crus I	R	**40 −60 −30**	***5.98***	**373**

Results were calculated at a voxel-threshold of p<0.001 (uncorrected) with a spatial extent of k≥31 voxels. Entries in bold denote activations significant at the FWE cluster-corrected level of p<0.05.

Underscored peak coordinates are given in Figure 3.

Percent signal changes of the BOLD response from the baseline (Figure 3) were chosen for the group local maxima in the conjunction activated regions for O-MI and O-IMIT, i.e. left PMv and right IPL. Figure 3 also diplays the percent changes in the BOLD signal for right OTC activated in all three conditions. All conditions showed activations higher than the baseline, with the exception of the O condition in the region of PMv. O-IMIT resulted in greater activity than the other two experimental conditions and O-MI had a greater activity than O except for OTC. ANOVA revealed a main effect of condition for PMv (F(2, 39) = 17.637; p<0.001) and IPL (F(2, 39) = 8.819; p<0.01), but not for OTC (F(2, 39) = 0.461; p = 0.662). In Bonferroni corrected post-hoc tests, O-MI and O-IMIT showed significantly greater increase in activation of PMv (p<0.05 and p<0.01) than O. Compared to O, O-IMIT additionally showed significantly greater activation in IPL (p<0.01).

### Head Motion

The analysis of the head motion parameters revealed a mean translation in the first run of −0.1 mm (SD = 0.17 mm) in the x-direction, +0.17 mm (SD = 0.23 mm) in the y-direction and +0.05 mm (SD = 0.32 mm) in the z-direction, and mean movements in the second run of +0.02 mm (SD = 0.24 mm) in the x-direction, 0.04 mm (SD = 0.31 mm) in the y-direction and 0.04 mm (SD ±0.51 mm) in the z-direction. None of the subjects’ head translation exceeded 2.5 mm in any direction.

Regarding the head rotation (degrees), a mean roll rotation of 0.003 (SD = 0.005) could be observed, mean pitch rotation was −0.002 (SD = 0.003) and the mean yaw rotation was −0.002 (SD = 0.002) in the 1^st^ run. In the 2^nd^ run, a mean roll rotation of −0.002 (SD = 0.009) could be observed, mean pitch rotation was −0.001 (SD = 0.003) and the mean yaw rotation was 0.001 (SD = 0.005). One subject (subject 8) showed an increased absolute rotation up to 0.04 in rolling, all others did not exceed 0.03 in rotation.

**Figure 4:**
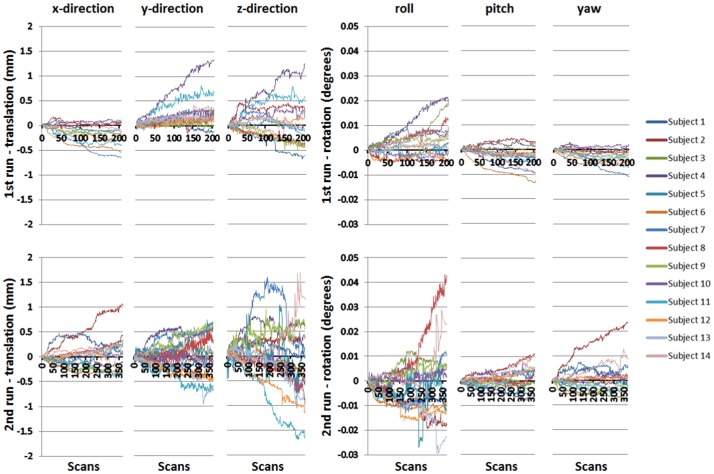
Head motion – Translation/Rotation. Left: Translation in x-, y- and z-direction of 14 subjects among 205 (1^st^ run - above) and 369 scans (2^nd^ run - below). Right: Rotations (roll, pitch, yaw) of 14 subjects among 205 (1^st^ run - above) and 369 scans (2^nd^ run - below). Regarding pitch rotation, the rotation is around the right-left-axis, moving the head up and down like shaking the head “yes”. Roll rotation is around the inferior-superior-axis, like shaking the head “no”. Yaw is rotation around the anterior-posterior-axis, like shaking the head “maybe”.

Single subject translations and rotations over the scanning period (1^st^ run and 2^nd^ run) are shown in Figure 4.

### Behavioral and EMG Assessment

The mean rating of the kinesthetic part of VMIQ was 2.1 (SD = 0.6) (1 =  image as vivid as normal vision; 5 =  no image at all) [40]. Hence, the imagined movements were on average very clear and vivid. Additionally, the subjects had to rate their subjective ability to imagine themselves performing simultaneously the observed foot movement (scale from 1 =  best to 5 =  worst). All reported no difficulty to imagine themselves in the position of the acting person displayed in the videos and controlling the foot movement. The mean rating was 1.6 (SD = 0.7). The correlation between the two ratings was highly significant (Pearson R = 0.731, p<0.05).

No significant EMG activity during O and O-MI was found in the right anterior tibialis muscle compared to the baseline. In contrast, the EMG activity significantly increased during O-IMIT when compared to the baseline (SCR), O and O-MI recordings (p<0.001).

## Discussion

In this study we compared the neuronal activation pattern revealed by observation of a goal-directed action (O) with that evoked when the subject simultaneously observes and imagines him/herself performing the action (O-MI). The control condition of O-MI was observation with online imitation (O-IMIT). The results confirmed our prediction that O-MI compared to O induced broader and stronger activations in the foot motor network. In addition, O-MI activated regions similar to those involved in O-IMIT.

### Online Motor Imagination (O-MI) Compared to Passive Observation (O)

In human the action observation network consists of a large group of brain regions involved in visual analysis of action and visuomotor performance [50]. In our investigation the simple observation (O) of a goal-directed foot movement yielded the most restricted activation pattern. Strong activations were mostly found in the visually processing posterior part of the PCu [51] and OTC regions, which transmit information from early visual areas [52] to parietal and PM components of the action observation network [50].

Apart from the left IPL, the foci in the right IPL and PM did not reach the statistical threshold during O-only and showed significantly less activation than in the other conditions. Although these results seem to surprise, previous investigations on observation of goal-directed hand movements did not consistently find activation in these regions [53]. In particular, studies on observation of transitive or intransitive foot movements reported only weak activations in these regions, even using less restrictive thresholds [23], [29]. While the reasons for these differences are unclear, it is interesting that using a forced-choice paradigm parietal and PM regions showed activation during observation of intransitive foot movements when attention was required [33]. In our investigation, the subjects were instructed to only watch the video, without being particularly attentive to the movements. In the study by Hotz-Boendermaker et al. [23] SCI patients activated bilaterally the PM cortex as compared to the unilateral activation in healthy subjects, suggesting that patients who are no longer able to execute foot movements need to focus their attention on the displayed foot movements more than healthy subjects. In general, vision can compensate for the increase in attention demands (e.g. [54]).

Compared to the O condition, O-MI showed consistently significant activation in IPL, PMv regions and putamen. When the two conditions were contrasted, the bilateral PMv and left IPL regions remained activated. These regions were also found in earlier studies we conducted using pure motor imagery (MI alone) in healthy subjects and spinal cord injury patients [12], [22]. In these earlier studies, alternating dorsal and plantar flexion of the right foot at a self-paced rhythm of approximately 0.5 Hz was either imagined alone (MI) or executed by the subjects. In addition the significantly reduced percent signal change in PMv for O compared with O-MI found in the present study is broadly in line with a recent study which reported that the brain areas involved in action observation of thumb-finger movements were a subset of those engaged in motor imagery with synchronous observation [10].

To our knowledge, imagination combined with observation has been investigated for dance sequences [8], intransitive thumb-finger [10], transitive hand movements [2] and gait [11]. These four publications revealed activation patterns similar to those of our present study, though with additional small activation foci in M1 and S1 in the first two studies. The involvement of these latter regions in motor imagery is very variable, as discussed in e.g. [55], [56], [57], [58]. Interestingly, Szameitat et al. [59] showed that activation during motor imagery best resembled that in motor execution in patients who had a stroke, but only second best in healthy subjects. Other studies support this finding [60], [61], [62], [63]. An explanation could be that patients need additional neural resources to execute a task. Therefore, observation of the movement during motor imagery might facilitate the imagination process. Another possible explanation could be an inhibitory effect of the presented transitive movement during observation. Villiger et al. [64] have shown that the excitability of the primary motor region was suppressed by observed transitive actions. This finding was confirmed later on by Hardwick et al. [65].

During the O-MI condition we also observed activation in PCu which has been previously reported during first-person motor imagery of walking in a virtual environment [38]. The PCu located in the mesial posterior cortex has been implicated in self-related mental representations and shown to be activated by visual-spatial imagination tasks and self-related stimuli [66], [67]. With respect to the anterior INS, activated when the O-MI was contrasted with O, the activation has been associated with several cognitive processes, such as attention and control of goal-directed tasks [68], [69], as well as awareness of causing an action, i.e. sense of agency [70]. Compared to our earlier studies with pure motor imagery, this region was the only region which was additionally detected.

### Control Condition: Online Imitation (O-IMIT) of a Transitive Foot Movement

The functional network activated during observation with synchronous imitation (O-IMIT) of transitive lower extremity movements has not yet been reported. We used this condition as a control, to test the role of execution in the activation patterns under the same conditions as O-MI. When contrasting O-IMIT with O, activations were similar to those reported for execution in our previous and other studies [22], [32], [71], i.e. M1/S1, SMA, PUT and CB.

To our knowledge, the only other investigations on first-person perspective imitation combined with observation of the foot movement are those of Chaminade et al. [16] and Jackson et al. [24]. Both studies included 5 s video clips of single hand or foot movements presented in first-person perspective. In the study by Chaminade et al. [16], the subjects were required to perform the presented hand or foot gestures, or to execute another gesture, either with the same or the other limb (hand or foot). They proposed that increased bilateral OTC activity was associated with increased visual attention and that visuo-spatial representation of the body was mainly sustained by the IPL. Jackson et al. [24] in addition addressed the question of perspective. The video clips were presented either in the first- or third-person perspective and the subjects either watched (observation) or imitated the actions in synchrony (imitation). They clearly showed that imitation during observation in the first-person perspective preferentially recruits motor regions, while in the third-person perspective activation was shifted towards visual areas. The activation patterns found in our study during the O-IMIT condition in the first-person perspective were comparable to the findings of the latter study, especially in M1/S1, PMv, IPL, PCu and CB. Therefore, the first-person perspective during O-IMIT may facilitate the integration of kinesthetic information and improve the reproduction of the action [24]. Interestingly, no obvious differences in perspective were reported when observation, imagination and imitation were investigated with thumb-finger movements [10]. This discordance in results may be explained by the recent findings of Caggiano et al. [72] who found view-invariant, as well as view-dependent cells, in the ventral premotor cortex (F5). If similar neuronal populations exist in humans, it would be very difficult to distinguish them using fMRI. Nevertheless, first-person perspective seems to have the strongest impact on the motor relevant network in fMRI, even in the presence of view-invariant cells.

### What is Common to O-MI and O-IMIT?

In previous publications by our group we reported that kinesthetic motor imagery and execution of foot movements are closely related and may be a continuum of one and the same phenomenon with just quantitative variations [22]. In the present study we show that observation with online motor imagination (O-MI) and online imitation (O-IMIT) are also strongly related, on the basis of the contrasts between the conditions, their conjunctions and the percent signal changes. The contrast between O-IMIT and O-MI revealed enhanced activation in SMA, right IPL, bilateral PUT and left CB and the conjunction of the two conditions uncovereda large number of shared regions, several of them belonging to the execution network, such as parietal and PM regions and ipsilateral CB [24], [29], [32]. Together with the lack of findings and the non-significant differences in percent signal changes in the contrast between O-MI and O-IMIT, this conjunction strongly suggests that the motor execution network can be engaged when the observation of an action is combined with simultaneous motor imagery, i.e. by the imagination that the action is performed by the subject. This conjunction also revealed activation in an extended part of the IPL, the temporal-parietal junction. This region has been shown to be involved in the perception of the self and of its interactions with the external world and therefore, to be a neural correlate of body ownership [70], [73], [74], [75], [76], [77], [78].According to the study by Macuga and Frey [7], observation with motor imagery activates a subset of the areas required for movement execution. In contrast to these findings, our experiments did not show significantly attenuated activation during O-MI compared to O-IMIT in selected regions, i.e.IPL and PM.

Taken together, the recruitment of similar brain regions during O-MI or O-IMIT could facilitate post-injury retaining of function and potentially promote functional recovery by increasing the activity of simple action observation – a subset of O-MI and O-IMIT. Thus, our findings may have clinical value in neurorehabilitation by guiding the observed actions when motor programs are still at least partly present, as it is often the case in stroke [71] or spinal cord injury patients [22]. An open and important question is how the reported activation patterns will change after long-term training in the condition O-MI and how their shaping over time will correlate with improved motor performance.

An important limitation of this experiment is that we did not include conditions with motor imagery only (MI) or imitation only (IMIT), i.e. without visual input. A follow-up experiment including these conditions would allow the comparisons O-MI vs MI and O-IMIT vs IMIT, revealing the additional effects that observation has on pure motor imagery and action imitation. A comparison with our existing conditions O vs O-MI and O vs O-IMIT would then provide a more complete picture of the relative individual contributions of O, MI and IMIT to the observed combined activation patterns.
